# RNF213 Acts as a Molecular Switch for Cav-1 Ubiquitination and Phosphorylation in Human Cells

**DOI:** 10.3390/cells14110775

**Published:** 2025-05-25

**Authors:** Jungmi Choi, Ryoichi Inoue, Yuki Masuo, Yukiko Shimizu, Kazuhiro Sonomura, Minsoo Kim, Hatasu Kobayashi, Kouji H. Harada, Yohei Mineharu, Akio Koizumi, Tohru Tezuka, Shohab Youssefian

**Affiliations:** 1 Laboratory of Molecular Biosciences, Graduate School of Medicine, Kyoto University, Yoshida-Konoe-cho, Sakyo-ku, Kyoto 606-8501, Japan; choijungmi.56z@st.kyoto-u.ac.jp (J.C.);; 2 Center for Genomic Medicine, Graduate School of Medicine, Kyoto University, Kyoto 606-8507, Japan; 3 Laboratory of Integrative Molecular Medicine, Graduate School of Medicine, Kyoto University, Kyoto 606-8501, Japan; 4 Department of Environmental and Molecular Medicine, Mie University Graduate School of Medicine, Tsu 514-8507, Japan; 5 Department of Health and Environmental Sciences, Graduate School of Medicine, Kyoto University, Kyoto 606-8601, Japan; 6 Department of Artificial Intelligence in Healthcare and Medicine, Kyoto University, Kyoto 606-8501, Japan; 7 Institute of Public Health, Kyoto Hokenkai Foundation, Kyoto 616-8149, Japan

**Keywords:** RNF213, moyamoya disease, MMD, caveolin-1, Cav-1

## Abstract

RNF213 encodes a unique protein with AAA+ ATPase and E3 ubiquitin ligase activities that are critical for its diverse roles, which range from involvement in human vasculopathies, such as Moyamoya disease, to ubiquitination of viral and bacterial pathogens. Nevertheless, its primary functions in human signaling remain unclear due to the limited identification of direct substrates. Here, we investigated the interaction between RNF213 and caveolin-1 (Cav-1), a small scaffolding protein vital for caveolae formation and the regulation of a plethora of cellular processes. Cav-1 specifically binds within the two functional AAA+ domains of RNF213 in an ATP-dependent manner, highlighting the influence of cellular energy status on this interaction. Consequently, RNF213 ubiquitinates Cav-1 at several N-terminal lysine residues through K48 and K63 linkages, although several Moyamoya disease-associated RNF213 mutations greatly reduce this polyubiquitination. Moreover, RNF213 activity inhibits phosphorylation of a key regulatory residue of Cav-1, as RNF213 knockdown under oxidative stress markedly enhances Cav-1 Tyr14 phosphorylation and modifies nitric oxide bioavailability in endothelial cells. Collectively, our results indicate that RNF213 functions as a molecular switch modulating Cav-1 signaling based on RNF213 functionality and cellular conditions. These findings offer new insights into vascular pathogenesis and the vast signal pathways along the RNF213–Cav-1 axis.

## 1. Introduction

Mutations in the RNF213 gene constitute major risk factors for Moyamoya disease (MMD), a rare idiopathic cerebrovascular disorder in which fine intracranial blood vessels develop as a result of vascular smooth muscle cell hyperplasia and subsequent steno-occlusive lesions of the internal carotid arteries [1,2,3,4]. These delicate and fragile compensatory blood vessels often lead to hemorrhagic strokes as well as other neurological disorders in patients. RNF213 mutations, especially the R4810K variant that is predominant in east Asian populations, have also recently been associated with other vasculopathies [4], including pulmonary [4,5,6], coronary [7], and renal [8,9]. In the case of pulmonary hypertension, RNF213 mutations have been implicated in abnormal vascular remodeling and endothelial dysfunction that contribute to a pathological increase in pulmonary arterial pressure and resistance [5,6]. While the exact etiology of these vasculopathies has not been clarified, they appear to be the result of a complex interplay between genetic predisposition and environmental stressors, such as hypoxia, inflammation, or oxidative stress [6,10].

RNF213 encodes a massive oligomeric protein (ring finger protein 213) consisting of 5207 amino acids with a molecular weight of 591 kDa. It includes two main modules: an AAA+ ATPase-containing domain responsible for ATP binding and hydrolysis, and an E3 module made up of RING and RZ finger domains that function as E3 ubiquitin ligases involved in auto- and substrate ubiquitination [11]. Of the six AAA+ domains in the dynein-like core of RNF213, only two are catalytically active and perform their ATPase functions in a manner analogous to dynein [11,12]. Such AAA+ ATPases form a distinctive group characterized by their doughnut-shaped complex formation, often comprised of hexamers [13,14], and their unique contribution to various intracellular physical processes, such as cargo transport, membrane dynamics, and protein complex dissociation [15]. The E3 ligase activities associated with the RING and the newly identified RZ finger in the E3 shell, together with several distinct ubiquitin-conjugating enzymes (E2), such as UBE2N and UBE2L3, are pivotal in the generation of K48- and K63-linked polyubiquitin chains on RNF213 itself [16,17], on bacterial and viral coats [18], and even on the lipid A moiety of bacterial lipopolysaccharides (LPSs) [19], leading to their proteasomal degradation or to downstream signaling events [18,19]. Despite the recent progress in identifying such pathogenic targets of RNF213, the identity of human substrates targeted for ubiquitination by RNF213 remains extremely limited [20,21], thus hampering progress in clarifying its direct in vivo functions and signal control mechanisms and its role in vasculopathies such as MMD.

Caveolin-1 (Cav-1), a 178-amino acid oligomeric cholesterol-binding membrane protein, plays a pivotal role in the formation and function of caveolae, small invaginations of the plasma membrane involved in a multitude of cellular processes [22,23,24]. These specialized membrane microdomains are involved in organizing and compartmentalizing signaling molecules, receptors, and lipids, and creating platforms for signal transduction and endocytosis [25,26]. Cav-1 is widely expressed in various cell types, including endothelial cells, fibroblasts, adipocytes, and smooth muscle cells [27,28]. In endothelial cells, for example, Cav-1 interacts directly with endothelial nitric oxide synthase (eNOS), inhibiting its activity and controlling nitric oxide (NO) production [29,30,31,32]. This Cav-1-eNOS interplay is crucial in maintaining vascular homeostasis, and its dysregulation has been implicated in the pathogenesis of various cardiovascular disorders [30,31,32]. In pulmonary hypertension, the dysregulation of Cav-1 contributes to abnormal vascular remodeling and increased pulmonary artery pressure, with a noted association between Cav-1 loss and disease progression [33,34].

Recent circumstantial evidence suggests functional links between RNF213 and Cav-1. Notably, Cav-1 levels are reduced in MMD patients carrying the RNF213 R4810K mutation [35], as well as in the lung tissue of RNF213 R4810K transgenic mice exposed to hypoxic stress [6]. In addition, both proteins have been associated with the progression of pulmonary arterial hypertension and with general vascular health [6,36], suggestive of some form of relationship that effects other vascular disorders. Furthermore, the expression of both RNF213 and Cav-1 in endothelial cells, the primary cell type implicated in RNF213-related vasculopathies, further implies that their interaction may be of biological significance. Finally, both Cav-1 and RNF213 have been implicated in cellular events that include endothelial cell dysfunction, lipid droplet formation, as well as lipid metabolism and signaling, cancer development, and even bacterial and viral endocytosis and pathogenesis [37,38].

Despite such epidemiological and basic research findings, the exact relationship between RNF213 and Cav-1 remains unclear. We hypothesized that Cav-1 and RNF213 may have more than just a causal relationship, and therefore, in this study, we investigated the direct interaction between RNF213 and Cav-1. Our novel findings of their interactions, and on the consequences of the ubiquitination and phosphorylation of Cav-1 by RNF213 on possible downstream signaling events, not only provide further insights into their functions but may also lead to the elucidation of the pathophysiological mechanisms underlying the various cellular events above as well as the onset and development of vasculopathies, such as MMD.

## 2. Materials and Methods

### 2.1. Cell Culture and Transfection

Human Umbilical Vein Endothelial Cells (HUVECs) and Human Pulmonary Artery Endothelial Cells (HPAECs) were purchased from Lonza (Lonza, Rockville, MD, USA) and used between passages 1–6. All experiments using HUVECs or HPAECs were conducted using a single donor-derived lot for each cell type. Cells were seeded onto type I collagen-coated plates (Iwaki, Fukushima, Japan) and cultured in endothelial basal media-2 (EBM-2, Lonza, USA) supplemented with growth factors and 2% FBS. Human embryonic kidney 293T (HEK293T, Dharmacon, Lafayette, CO, USA) cells were cultured in Dulbecco’s modified Eagle’s medium (Nacalai Tesque, Kyoto, Japan) containing 10% fetal bovine serum (Thermo Fisher Scientific, Waltham, MA, USA) and 100 units/mL penicillin (Nacalai Tesque, Japan). Cells were maintained at 37 °C in a humidified, 5% CO_2_, and atmospheric air-balanced chamber. Media were changed every 2 days, and cells were passaged when 80–90% confluent. Cells were transfected with plasmids using Viofectin (Viogen Bio Tek, Taipei, Taiwan) or with siRNA using Lipofectamine™ RNAiMAX Transfection Reagent (Invitrogen, Carlsbad, CA, USA, Thermo Fisher Scientific, USA) as described in the manufacturer’s protocols.

### 2.2. Human Clones and Plasmids

3xFLAG-RNF213 in pcDNA3 was from our previous studies [16,39], and HA-Cav-1 in pCMV3 was purchased from Sino Biological (Sino Biological, Beijing, China). The RNF213 deletion clones N1, N2, C3, and C5 were kindly provided by Dr. T. Habu (Mukogawa Women’s University). All other deletions, point mutations, or other modifications to RNF213, Cav-1, and their associated tags were either obtained from our previous studies [16,40] or developed in the current study by PCR-based mutagenesis and cloning using the In-Fusion HD cloning system (Clontech/Takara, Shiga, Japan). Human ZNRF1 cDNA in pOTB7 was purchased from RIKEN (BRC Cell Bank, Ibaraki, Japan) and recloned as 3xFLAG-ZNRF1 in pcDNA3. Myc-ubiquitin was generated in pcDNA3. All DNA modifications were confirmed by DNA sequence analysis.

### 2.3. siRNA-Directed Gene Silencing

Knockdown of RNF213 and/or Cav-1 in HUVECs and HPAECs was performed using 10 or 20 nM siRNAs. For RNF213 knockdown, we used siRNAs targeting three different RNF213 sequences, i.e., (1) CGtAtGAGCtCACAACCGA (Sigma-Aldrich, St. Louis, MO, USA), (2 and 3) GGACtGGtGCCtttGCAtt (Sigma-Aldrich, USA and Invitrogen, USA, respectively), and (4) CAGCtGCtGtGAAAAACGA ([41], Sigma-Aldrich, USA). For Cav-1 knockdown, we used three pooled siRNA sequences, i.e., (1) CCAAGttGCtAAtACAGCAA, (2) CCCtAAACACCtCAACGAt, and (3) CCttCACtGtGACGAAAtA (Sigma-Aldrich, USA). A non-targeting universal scramble siRNA (Sigma-Aldrich, USA) was used as the negative control. Dose responses for Cav-1 and RNF213 siRNA were examined and the knockdown effects validated by Western blot between 24 h and 48 h post-transfection. Cells were harvested at various time points following transfection based on the specific assay.

### 2.4. Immunoprecipitation

Following transfection and/or exposure to treatment, cells were washed and scraped off the plate wells with ice-cold 1 × PBS. After pelleting the cells at 1000× *g* for 5 min, PBS was completely removed and cells were lysed in ice-cold lysis buffer containing 150 mM NaCl, 50 mM Tris-HCl pH 7.6, 2 mM EDTA, 1% NP-40, and a 1 × protease inhibitor cocktail (Nacalai Tesque, Japan) for 30 min at 4 °C on a rotator. To prevent post-lysis Cav-1 oligomerization, cell lysis and sample handling were performed under optimized conditions using mild detergents and at 4 °C [42]. Lysates were centrifuged at 10,000× *g* for 15 min at 4 °C and the supernatants frozen and kept at −80 °C until use. Protein concentrations were determined using the Bradford assay (Bio-Rad, Hercules, CA, USA). Cellular extracts were incubated with the indicated antibody overnight, followed by a 1 h incubation with pre-washed Protein A/G beads (Millipore, Burlington, MA, USA) at 4 °C. Cellular extracts and Protein A/G beads were separated by centrifugation at 5000× rpm for 1 min at 4 °C. The beads were washed five times with lysis buffer, and the remaining Protein A/G beads pellets were resuspended in 6 × SDS loading buffer and boiled for 10 min at 99 °C. For TUBE assays, used to confirm specific ubiquitin linkages, cellular extracts were incubated overnight with pre-washed K48 or K63 TUBE biotin beads (LifeSensors, Malvern, PA, USA), followed by centrifugation at 5000× rpm for 1 min at 4 °C to precipitate the biotin beads, which were then washed five times with lysis buffer. The final bead pellets were resuspended in 6 × SDS loading buffer and boiled for 10 min at 99 °C.

### 2.5. Immunoblotting

Cell lysates (10–20 μg per lane) were separated by 5–20% SDS-PAGE (Nacalai Tesque, Kyoto, Japan) with known molecular weight markers (Bio-Rad, USA) and transferred onto polyvinylidene fluoride membranes (Merck KGaA, Darmstadt, Germany) by standard procedures. Membranes were incubated in blocking solution (Blocking One, Nacalai Tesque, Japan) for 3 h at room temperature and hybridized overnight at 4 °C with primary antibodies at appropriate dilutions. Membranes were washed three times with TBS-T and then incubated with either anti-rabbit or anti-mouse (1:10,000) (Sigma-Aldrich, USA) HRP-labeled secondary antibodies for 1 h at room temperature. Chemiluminescent signals were developed by ECL Prime (GE Healthcare, Chicago, IL, USA) or Chemi-Lumi One (Nacalai Tesque, Japan), analyzed using an LAS3000 imager (FUJIFILM, Tokyo, Japan), and quantified using ImageJ software 1.51 (NIH, Bethesda, MD, USA). Exposure times were optimized to avoid signal saturation, and all quantifications were based on non-saturated levels to ensure that the densitometric analyses were performed in the linear response range. The primary antibodies used in this study are listed in Supplementary Table S1.

### 2.6. Cell Imaging

HUVECs and HPAECs were grown on glass coverslips, fixed with 4% paraformaldehyde at room temperature for 15 min, incubated in PBS(+) with 0.3% Triton X-100 at room temperature for 15 min, blocked in PBS(+) with 5% FCS at room temperature for 2 h to minimize nonspecific binding, and incubated with anti-RNF213 or anti-Cav-1 antibodies overnight at 4 °C. Washed slides were incubated with secondary antibodies for 1 h at room temperature with rabbit polyclonal goat anti-rabbit IgG labeled with Alexa Fluor™ Plus 555 (A32732, Thermo Fisher Scientific, USA) for RNF213, and goat anti-mouse IgG labeled with Alexa Fluor™ Plus 488 (A32723, Thermo Fisher Scientific, USA) for Cav-1 for 30 min. Coverslips were mounted in Mowiol (Sigma-Aldrich, USA) containing 0.5 μg/mL DAPI (Nacalai Tesque, Japan). Imaging was performed on a confocal laser scanning microscope (FV1000-D IX81 or FV3000-OSR, Olympus, Tokyo, Japan) with a 63× oil immersion objective. The pinhole size was set to 1 Airy unit, resulting in an optical section thickness of approximately 0.8 μm under the default system settings. In control experiments, secondary antibody-only staining was performed to confirm signal specificity. The anti-Cav-1 antibody used has been previously validated for immunofluorescence microscopy [43,44], and the anti-RNF213 antibody has also been used successfully in immunofluorescence analysis [45].

### 2.7. Depletion and Measurement of Intracellular ATP Levels

Intracellular ATP levels in HEK293T cells were depleted using a basic mixture of 10 mM 2-deoxy-D-glucose (2DG, TCI, Englewood, CO, USA) and 10 mM sodium azide (NaN_3_, FUJIFILM Wako Pure Chemical Corporation, Osaka, Japan), or dilutions of these, for 1 h [46,47]. To determine cellular ATP levels, cells were lysed in mammalian cell lysis buffer, mixed with substrate solution according to the manufacturer’s instructions (ATP-lite, PerkinElmer, Waltham, MA, USA), and 100 μL aliquots then used to determine the luminescence levels against standard ATP amounts in white non-phosphorescent 96-well plates using a Wallac multi-label microplate reader (Arvo SX 1420, PerkinElmer, USA).

### 2.8. Mass Spectrometry

RNF213 and Cav-1, each tagged with either 3xFLAG or HA, were transfected into HEK293T cells, and the cells were grown until 90% confluence. Cav-1 and its ubiquitinated forms were enriched from cell lysates by immunoprecipitation using an anti-Cav-1 antibody [48]. The eluted immunoprecipitated products, containing both monomeric and oligomeric forms of Cav-1, were subjected to a brief separation by SDS-PAGE on a 5–20% gradient gel. Gel lanes, containing the Cav-1-ubiquitinated protein complexes, were isolated, cut into 2 mm slices using an automatic gel slicer, and then subjected to in-gel reduction with dithiothreitol and subsequent destaining and drying by vacuum centrifugation. Protein was reduced and S-alkylated with dithiothreitol and iodoacetamide, respectively, followed by trypsin digestion in 25 mM NH_4_HCO_3_ at 37 °C overnight. Digested peptides were extracted and cleaned with a GL-Tip SDB (GL Sciences, Tokyo, Japan), and the purified peptide samples were injected into a NanoLC Orbitrap fusion system (Thermo Fisher Scientific, USA). Peptides were separated on NTCC-360 columns (125 mm length × 75 μm ID, C18 particle diameter 3 μm, Nikkyo Technos, Tokyo, Japan). The injection volume was 5 μL, and the flow rate was 500 nl/min. The mobile phases were (A) 0.1% formic acid and (B) 0.1% formic acid and 80% acetonitrile. The gradient program was as follows: 0–3 min, 5% B; 3–90 min, linear gradient to 40% B; 90–105 min, 99% B; 105–130 min, 5% B. The mass scan range was *m/z* 350 to 1650, with a maximum injection time of 50 msec and detection at a mass resolution of 60,000 in the Orbitrap analyzer. The top ten precursor ions with +2, +3, or +4 charge were selected in each MS scan for a subsequent MS/MS scan. The normalized higher energy collisional dissociation was set at 30, with detection at a mass resolution of 15,000 in the Orbitrap analyzer. The obtained data were analyzed with Proteome Discoverer 2.0 software (Thermo Fisher Scientific, Waltham, MA, USA), and the peptides containing glycylglycine modification at lysine residues were identified.

### 2.9. NF-κB Dual Luciferase Assays

HEK293T cells were transfected with various combinations of the following plasmids: firefly luciferase expression plasmid driven by NF-κB-responsive elements (pGL4.32, Promega, Madison, WI, USA), Renilla luciferase expression plasmid driven by the HSV thymidine kinase promoter (pGL4.74) as an expression control, and pcDNA3.1 empty vector, HA-Cav-1, or 3×FLAG-RNF213 expression plasmids. Cells were lysed 40 h post-transfection and subjected to dual-luciferase reporter assays (Promega, USA) using a multi-label microplate reader (Arvo SX 1420, PerkinElmer, USA).

### 2.10. HPAEC H_2_O_2_ Treatments and Nitric Oxide (NO) Measurements in Live Cells

HPAECs were grown to 80% confluence in EBM-2 as above, transfected without any siRNA, with scramble siRNA, or with siRNAs targeting RNF213, Cav-1, or both RNF213 and Cav-1. After growth for an additional 48 h, cells were treated with 1 mM H_2_O_2_ for 30 min to induce oxidative stress, and then immediately harvested, lysed, and used for Western blot analysis as above. NO production in live HPAECs was determined using the dye DAF-FM DA (4-Amino-5-Methylamino-2′,7′-Difluorofluorescein Diacetate, Goryo Chemical, Sapporo, Japan) according to the manufacturer’s protocol. Briefly, HPAECs were seeded at 3.5 × 10^3^ cells per well in 96-well collagen-coated plates, maintained in growth media overnight, and then transfected with scramble siRNA or siRNAs targeting RNF213, Cav-1, or both. Transfected HPAECs were then treated with 10 μM DAF-FM DA for 30 min, followed by the addition of 1 mM of H_2_O_2_. Snapshots of the live cells were captured every 5 min over a 30 min period using a fluorescent microscope with a 495 nm optimum excitation wavelength and 515 nm fluorescence wavelength (FV3000, Olympus, Japan). For analysis, regions of interest were established at the site of observation, and the average fluorescence intensity in that area was obtained using ImageJ software 1.51 (NIH, USA).

### 2.11. Statistical Analysis

Results are presented as the mean ± standard error of the mean (SEM). Data were first tested for normality using the Shapiro–Wilk test. Differences between more than two samples were tested using one-way or two-way analysis of variance (ANOVA). When ANOVA was significant, i.e., *p* < 0.05, then Tukey’s honestly significant difference test was conducted to detect different groups. For data normalized to wild-type (WT = 1.0), one-sample *t*-tests were performed to determine whether the mean of each mutant group significantly differed from the baseline value of 1.0. When multiple one-sample *t*-tests were performed, *p*-values were adjusted for multiple comparisons using the Holm–Šídák correction method. All statistical analyses were conducted using Prism 9 software (GraphPad, USA). The following symbols were used for the respective significance levels: * *p* < 0.05, ** *p* < 0.01, *** *p* < 0.001, **** *p* < 0.0001. 

## 3. Results

### 3.1. RNF213 Co-Localizes and Interacts with Cav-1 in Endothelial Cells

**Figure 1:**
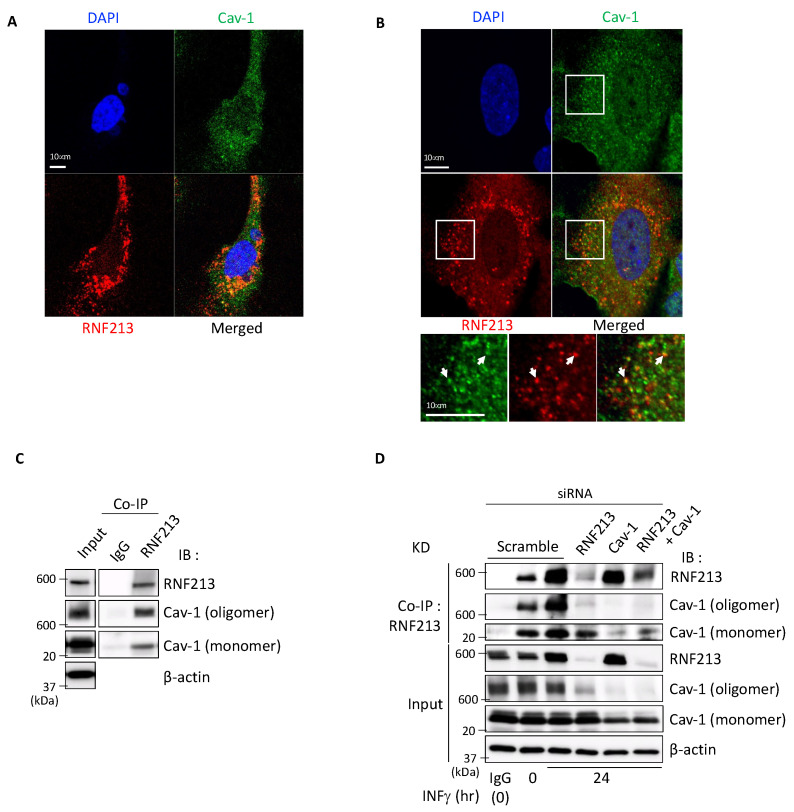
RNF213 co-localizes and interacts with Cav-1 in endothelial cells. (**A**) Confocal immunofluorescence microscopy using HUVECs, and (**B**) super-resolution confocal microscopy with HPAECs (white boxes represent magnified regions shown below). In both cases, nuclei were stained with DAPI (blue), Cav-1 was identified with Alexa-488 (green), and RNF213 was tagged with Alexa-555 (red). In the merged figures and inset, orange and yellow locations indicate co-localization of RNF213 and Cav-1 (white arrowheads). (**C**) Co-immunoprecipitation (co-IP) assay with an IgG negative control or RNF213 antibody from HUVEC lysates, followed by immunoblot (IB) analysis with the indicated antibodies. (**D**) Co-IP with an IgG control or RNF213 from HPAEC lysates transfected with scramble siRNA or siRNAs targeting RNF213, Cav-1, or both RNF213 and Cav-1, and treated with 100 ng mL^−1^ INFγ for 0 or 24 h to enhance RNF213 protein levels, followed by IB analysis with the indicated antibodies. The detected protein sizes are RNF213 (591 kDa), Cav-1 oligomer (>600 kDa) and Cav-1 monomer (~24 kDa). All analyses involving HUVECs and HPAECs were performed using a single donor-derived lot for each cell type, with a minimum of three independent experimental replicates to ensure consistency and reproducibility.

As a first step in determining the possible interactions between RNF213 and Cav-1, we analyzed their subcellular localizations in endothelial cells, which express both RNF213 and Cav-1, and which are considered as the primary targets of RNF213-related vasculopathies [49,50,51,52]. Confocal immunofluorescence microscopy of HUVECs showed that both RNF213 and Cav-1 have a diffused and particulate cytoplasmic distribution with some areas of overlap, as shown by the yellow- and orange-stained locations in the merged image (Figure 1A), indicative of their partial co-localization. To obtain a more detailed visualization, we used super-resolution confocal microscopy of HPAECs, and found that the enhanced resolution confirmed a more definitive co-localization between RNF213 and Cav-1, as demonstrated by distinct areas of yellow staining (white arrows) in the merged image (Figure 1B and inset).

Interactions between RNF213 and Cav-1 were then investigated by co-immunoprecipitation assays (co-IP) using HUVEC and HPAEC lysates. In HUVECs, immunoprecipitation with RNF213, but not with an IgG negative control, successfully co-immunoprecipitated both the monomeric (~22 kDa) and oligomeric (>600 kDa) forms of Cav-1 [53], indicative of a physical association between the two endogenous proteins (Figure 1C). Similarly, in HPAECs, treated with IFNγ to enhance RNF213 expression [39], the clear interactions between RNF213 and Cav-1 could be greatly reduced by siRNA-mediated knockdown of RNF213, Cav-1, or both RNF213 and Cav-1 (Figure 1D). The extent of siRNA-mediated knockdown in these samples was approximately 95% for RNF213, 40% for monomeric Cav-1, and 90% for oligomeric Cav-1. When RNF213 and Cav-1 were knocked down simultaneously, the knockdown efficiencies were 94% for RNF213, 40% for monomeric Cav-1, and 91% for oligomeric Cav-1. These knockdown experiments confirm the identities of the expressed RNF213 and Cav-1, and together provide clear initial evidence that a sub-population of RNF213 and Cav-1 co-localize and physically interact within these endothelial cells.

### 3.2. Cav-1 Specifically Binds Within the Functional AAA+ Domains of RNF213

**Figure 2:**
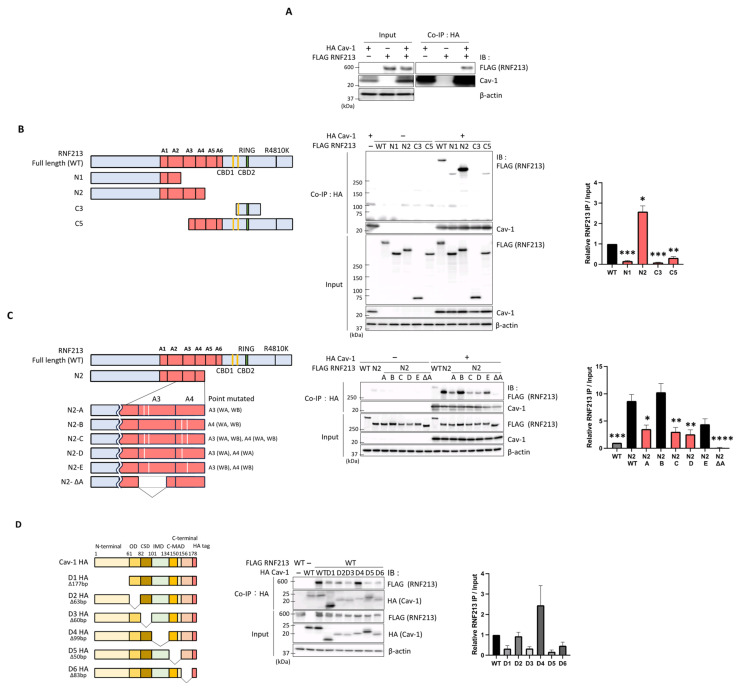
Identification of the RNF213 and Cav-1 domains necessary for their interaction. (**A**) HEK293T cells were co-transfected with combinations of FLAG-tagged full-length (WT) RNF213 and HA-tagged Cav-1 as indicated. Interactions between RNF213 and monomeric Cav-1 were detected by co-immunoprecipitation (co-IP) with anti-HA antibody, followed by immunoblot (IB) analysis with the indicated antibodies. (**B**) Schematic diagram of FLAG-tagged WT RNF213 and four different truncated RNF213 fragments (N1, N2, C3, and C5), and their use in co-IP experiments in HEK293T cells also overexpressing HA-tagged Cav-1. Co-IP with anti-HA antibodies followed by IB with the indicated antibodies were performed to identify the RNF213 regions interacting with monomeric Cav-1. (**Right panel**) The ratios of bound RNF213 to total input RNF213 were calculated (mean ± SEM, *n* = 3), with the mean WT ratio being arbitrarily defined as 1. Asterisks denote significant statistical difference from the WT, determined by one-sample *t*-tests with Holm–Šídák correction for multiple comparisons (* *p* < 0.05, ** *p* < 0.01, and *** *p* < 0.001). (**C**) Schematic diagram of WT RNF213, the N2 fragment, and the various N2 fragments with point mutations in the Walker A (WA) and/or Walker B (WB) of the A3 and/or A4 domains, or a complete deletion of the WA and WB region (ΔA) of the A3 domain of RNF213. “ΔA” refers to an internal deletion mutant of RNF213, in which amino acids 2365–2613 encompassing the A3 AAA+ domain [16] were deleted to assess the functional contributions of this region. Co-IP experiments were performed using FLAG-tagged RNF213 fragments and HA-tagged Cav-1 overexpressed in HEK293T cells, following the same co-IP procedure as in (**B**), to identify the RNF213 domains essential for binding Cav-1. (**Right panel**) The ratios of bound RNF213 to total input RNF213 were calculated (mean ± SEM, *n* = 4), with the mean WT ratio being arbitrarily defined as 1. Asterisks denote significant statistical difference from the N2 WT by one-way ANOVA with * *p* < 0.05, ** *p* < 0.01, *** *p* < 0.001 and **** *p* < 0.0001. (**D**) Schematic diagram of full-length Cav-1 and six truncated forms with serial deletions of domains D1–D6 (see Section 3 for full description). HEK293T cells were co-transfected with FLAG-tagged WT RNF213 and HA-tagged full-length or deleted Cav-1 fragments as above, followed by co-IP as in (**B**). (**Right panel**) The ratios of bound RNF213 to total input RNF213 were calculated (mean ± SEM, *n* = 3), with the mean WT Cav-1 ratio being arbitrarily defined as 1. For all immunoblots, β-actin was used as the protein loading control. All experiments were performed with a minimum of three independent experimental replicates to ensure consistency and reproducibility.

To understand more about the interaction between RNF213 and Cav-1, and thus about their individual functions, we then sought to identify the exact sites of their interaction. However, as endothelial cells already express high levels of RNF213 and Cav-1, and transfecting them with modified DNA would be both confounding and technically challenging, we performed the binding assays in the easily transfectable HEK293T cells that express very low levels of these two proteins. Co-expression of FLAG-tagged RNF213 with HA-tagged Cav-1 in these HEK293T cells consistently demonstrated the co-IP of RNF213 and Cav-1 as in our HUVEC and HPAEC lines (Figure 2A).

At first, we focused on regions in RNF213 essential for binding Cav-1. Canonical caveolin-binding motifs (CBMs) [22] are characterized by the specific arrangement of aromatic (Φ) amino acids (such as ΦXΦXXXXΦ and ΦXXXXΦXXΦ), and are generally recognized as critical domains in various proteins essential for binding Cav-1 and thereby regulating numerous cellular processes [54]. We determined that RNF213 possesses two such putative CBMs (CBM1 and CBM2), between the AAA+ module and the RING domain, from positions 3711 and 3813, respectively. To examine their role in Cav-1 binding, we constructed point mutations in one or both CBMs of the RNF213-expressing plasmids, replacing the essential phenylalanine (F), tryptophan (W), and tyrosine (Y) aromatic residues with alanine (A) residues (Supplementary Figure S1A left). Co-IP of HEK293T cells transfected with WT or mutated RNF213 and Cav-1 unexpectedly revealed that mutation of all three residues in these putative CBMs did not reduce or alter the RNF213-binding affinities (Supplementary Figure S1A right), indicating that these regions of RNF213 are not involved in its interactions with Cav-1.

To identify the actual RNF213 domain(s) responsible for binding Cav-1, we first used four RNF213 deletion mutants: N1, N2, C3, and C5 (Figure 2B left). Co-IP experiments demonstrated that fragments N1 (containing the N-terminal and A1 and part of the A2 AAA+ domains), C3 (with only a region surrounding the RING domain), and C5 (lacking the N-terminal and A1–A3 AAA+ domains) showed significantly lower Cav-1 binding that the WT RNF213, whereas the N2 fragment (expressing the N-terminal and the A1–A4 AAA+ domains) displayed significantly higher Cav-1 binding than the WT protein (Figure 2B right).

To further specify the Cav-1-binding region around the N2 fragment of RNF213, we generated the N3, N6, N7, N8, N9, ΔA, N9-1, N9-2, N9-5, and N9-6 deletion fragments (Supplementary Figure S1B). In brief, the short N3 fragment (including only the functional A3 and A4 AAA+ domains) showed higher binding than the WT but less than the N2 fragment; the N6 and N7 fragments containing the IR3 and IR5 sites that have been proposed to serve as ATP-binding inhibitory sites [11] and the N8 fragment lacking only the IR5 site displayed considerably reduced Cav-1 binding, whereas the N9 fragment lacking a larger area around the IR5 site and containing only the IR3 site had relatively higher binding but less than N2; and the ΔA fragment, in which the functional domains of A3 AAA+ were completely deleted, had almost no binding compared to WT (Supplementary Figure S1C). Finally, as the N9 fragment lacked the region upstream of the AAA+ domain present in N2 and had relatively lower binding, further co-IP assays with the N9-1, N9-2, N9-5, and N9-6 fragments suggested that a short region upstream of the A1 domain, contained in fragments N9-1 and N9-2 but lacking in N9-5 and N9-6, could also enhance Cav-1 binding (Supplementary Figure S1D). Taken together, these findings reveal that site(s) within the A3 and A4 AAA+ domains are essential for the highest level of Cav-1 binding, but that downstream inhibitory regions may reduce this binding, and regions upstream of A1 may promote further Cav-1 binding.

Each of the active AAA+ domains is known to possess a Walker A (P-loop) and Walker B motif, essential for ATP binding and ATP hydrolysis, respectively [55]. To determine which of these specific motifs in A3 and A4 are important for Cav-1 binding, we constructed point mutations in each of these sequences, i.e., mutation of the nucleotide-binding K residue to an A residue in the consensus G-x(4)-GK-TS Walker A (WA) sequence and/or mutation of the nucleotide-hydrolyzing E residue to an A residue in the consensus hhhDE Walker B (WB) sequence (Figure 2C left). Co-IP assays with Cav-1 showed that the N2-A fragment with both Walker A and Walker B mutations in the A3 domain, the N2-C fragment with mutations in all four sites of the A3 and A4 domains, and the N2-D fragment with only Walker A mutations in both the A3 and A4 domains had significantly reduced Cav-1 binding compared to the WT RNF213 N2 fragment. However, the N2-B fragment with both Walker A and Walker B mutations in the A4 domain, and the N2-E fragment with mutations only in the Walker B of both A3 and A4, showed equivalent or non-significant reductions in Cav-1 binding compared to the N2 fragment (Figure 2C right). Overall, these findings, together with the data from the ΔA fragment in which the A3 domain of N2 was deleted and showed almost no Cav-1 binding, strongly suggest that the Walker A region in the A3 AAA+ domain of RNF213, which is necessary for ATP binding, is also essential for binding Cav-1. However, further detailed analyses are required to determine the extent to which ATP hydrolysis also contributes to this process.

Next, to investigate the specific domains in Cav-1 necessary for binding RNF213, we generated Cav-1 deletion mutants and examined their interactions with RNF213. The mutants included D1, lacking the N-terminal domain (amino acids (aa) 1–60); D2, lacking the upstream of oligomerization domain (OD, aa 61-81); D3, lacking the scaffolding domain (CSD, aa 82-101); D4, lacking the intramembrane domain (IMD, aa102-134); D5, lacking the C-terminal membrane attachment domain (C-MAD, aa 135-156); and D6, lacking the C-terminal domain (aa 157-178) [56,57] (Figure 2D left). Co-IP assays demonstrated that the deletion of none of these domains alone resulted in a complete loss of RNF213 binding, although binding was generally reduced in most fragments (Figure 2D right). Interestingly, in the D4 fragment lacking the IMD, RNF213 binding was even higher than that of Cav-1 WT. This hydrophobic hairpin loop transmembrane domain is inserted into the cytoplasmic face of the plasma membrane that segments the carboxy- and amino-termini and makes both cytoplasmic. Therefore, the IMD deletion presumably localizes Cav-1 to the cytosol, and favors binding to cytoplasmic RNF213. These results indicate that Cav-1 most probably interacts via several of its domains with RNF213.

Finally, we evaluated the effect of modifying the Cav-1 tyrosine-14 (Y14) phosphorylation site essential for Cav-1 activation or the caveolin-scaffolding domain (CSD) site necessary for target protein binding on the interactions of Cav-1 with RNF213. Mutations of Y14 to the phosphomimetic Y14D (YD) or to the phosphodefective Y14F (YF) forms, or those converting the FTVT protein-binding domain of the CSD to the inactive ATAT (FV) form had no effect on the binding of Cav-1 to RNF213 (Supplementary Figure S1E). These results suggest that neither the phosphorylation status of the Y14 site nor the CSD of Cav-1 play a particular role in its interactions with RNF213.

### 3.3. Interactions Between RNF213 and Cav-1 Are ATP-Dependent

**Figure 3:**
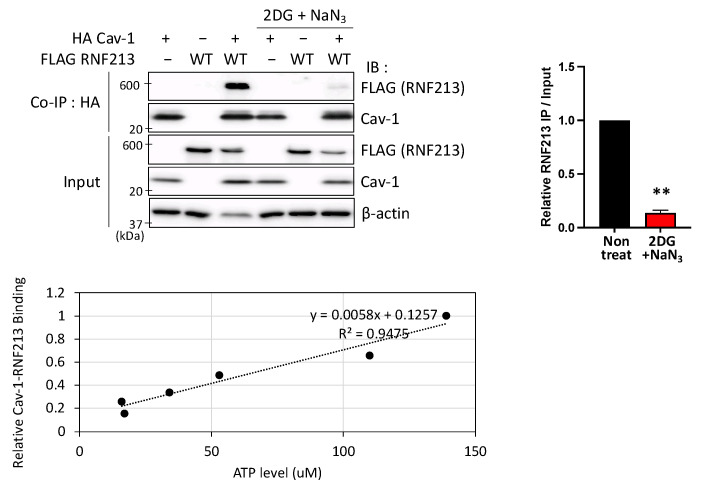
RNF213 binding to Cav-1 is ATP-dependent. Co-immunoprecipitation (co-IP) experiments using HEK293T cells overexpressing full-length FLAG-tagged WT RNF213 and HA-tagged Cav-1 under varying intracellular ATP concentrations and their effects on RNF213 and monomeric Cav-1 interactions. Cells were treated with 10 mM 2DG and 10 mM NaN_3_ for 30 min to modulate intracellular ATP levels, and the samples were then used for co-IP with anti-HA antibody, followed by immunoblotting (IB) with the indicated antibodies (**upper panel**). β-actin was used as the protein loading control. (**Right panel**) The ratios of bound RNF213 to total input RNF213 were calculated (mean ± SEM, *n* = 3), with the mean non-treatment control ratio being arbitrarily defined as 1. Asterisks denote significant statistical difference from the non-treatment control by one-sample *t*-tests with ** *p* < 0.01. Intracellular ATP levels were assessed using a luminescence-based ATP assay. The intracellular ATP levels were finely modulated by altering the levels of the inhibitors used, and the relationship between cellular ATP levels and the interactions between RNF213 and Cav-1 is shown by the regression line with an *R*^2^ value of 0.9475 (**lower panel**). The reference value of 1 on the *Y*-axis corresponds to 150.74 µM. Experiments were performed with three independent experimental replicates to ensure consistency and reproducibility.

Having identified that the RNF213 Walker A motif of the A3 AAA+ domain, which is one of the two active AAAs of RNF213 that require ATP binding for activity [11], is necessary for Cav-1 binding, we investigated the relationship between intracellular ATP availability and RNF213 binding of Cav-1. We modulated the intracellular ATP levels in HEK293T cells, transiently overexpressing RNF213 and Cav-1, with the standard method of using the glycolytic inhibitor, 2-deoxy-D-glucose (2DG), together with the oxidative phosphorylation inhibitor, sodium azide (NaN_3_). ATP levels were quantified using a luminescence-based assay using firefly luciferase, a sensor of intracellular ATP levels, and co-IP experiments were simultaneously performed. While Cav-1 was able to effectively pull down RNF213 under standard conditions, there was only negligible binding when the cells were treated with the inhibitors that reduced standard intracellular ATP levels by 80% (Figure 3). Furthermore, when we finely modulated the intracellular ATP levels by the level of inhibitors, we found a direct correlation between the ATP levels and the binding of RNF213 and Cav-1, with an *R*^2^ value of 0.9475. This strong correlation confirms that the extent of binding between RNF213 and Cav-1 is dependent on intracellular ATP availability and, thus, possibly on the potential of the RNF213 Walker A motif of the A3 AAA+ domain to bind ATP.

### 3.4. Cav-1 Suppresses Enhanced NF-κB Activity and Apoptosis Induced by RNF213 RING Mutations

**Figure 4:**
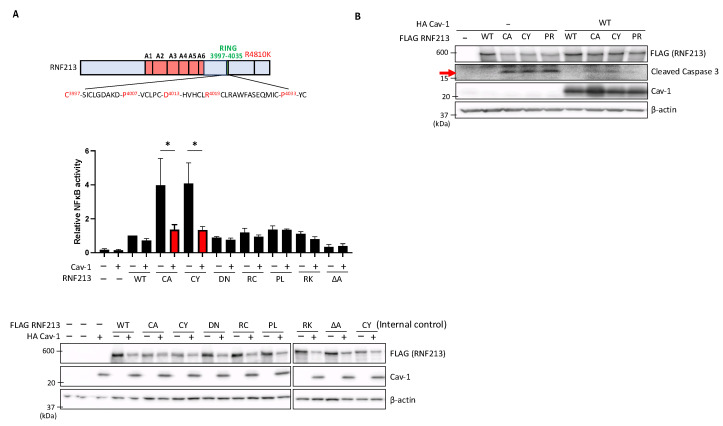
Cav-1 suppresses enhanced NF-κB activation and apoptosis induced by RNF213 RING mutations in HEK293T cells. (**A**) HEK293T cells were co-transfected with firefly and Renilla luciferase constructs as well as with FLAG-tagged WT or mutant RNF213, including C3997A (CA), C3997Y (CY), D4013N (DN), R4019C (RC), P4033L (PL), R4810K (RK), and the third AAA+ deletion (ΔA), or with the pcDNA3.1 empty vector as control (EV), together with and without HA-tagged Cav-1. Lysed cells were then used for dual-luciferase reporter assays. Data are normalized to Renilla luciferase (mean ± SEM, *n* = 3). Asterisks (and red columns) denote significant statistical differences in a one-way ANOVA test with * *p* < 0.05. (Lower panel) Immunoblot analysis showing expression levels of FLAG-tagged WT and mutant RNF213 with and without HA-tagged Cav-1 co-transfection used in the luciferase assays. The CY samples shown on both the left and right panels are the same and were included as internal controls to allow comparison across membranes. (**B**) HEK293T cells were co-transfected with FLAG-tagged WT or mutant RNF213, together with and without HA-tagged WT Cav-1. The levels of cleaved-Caspase-3 induced by the mutants and suppressed by Cav-1 were then determined by immunoblot analysis. The arrow shows the specific cleaved-Caspase-3 bands of interest. All experiments were performed with a minimum of three independent experimental replicates to ensure consistency and reproducibility. For all immunoblots, β-actin was used as the protein loading control.

In our previous study, we reported that specific mutations in the RING domain of RNF213 could enhance NF-κB activation and apoptosis of the transfected cells [16]. Here, therefore, we investigated whether co-expression of Cav-1 in these HEK293T cells could, by interacting with RNF213, affect these processes induced by the specific RING mutations. We conducted luciferase assays using an NF-κB reporter system, and confirmed our previous findings that NF-κB activity is slightly elevated above empty vector (EV) levels by expression of WT RNF213 or the D4013N and R4019C mutations, but greatly elevated by the RNF213 RING-dead C3997A and C3997Y mutations that disrupt coordination of RING zinc ions essential for domain integrity and E3 ligase activity, and effectively renders them as RING-dead. However, we now find that co-expression of Cav-1 in these HEK293T cells significantly suppressed the NF-κB activation by the C3997A and C3997Y mutations (Figure 4A). Moreover, to assess the impact of Cav-1 overexpression on mutant RNF213-induced apoptosis, we examined the levels of cleaved-Caspase-3, a key player in apoptotic pathways. As in our previous study [16], we found that the C3997A, C3997Y, and P4007R mutations greatly increased the cellular cleaved-Caspase-3 levels, whereas co-expression of Cav-1 effectively suppressed these increases (Figure 4B). Together, these findings suggest that Cav-1, presumably through its interaction within the two active AAA+ domains of RNF213, exerts a regulatory influence on the activation of NF-κB and apoptosis induced by mutations in the RNF213 RING domain.

### 3.5. RNF213 Mediates the Ubiquitination of Cav-1 at Multiple Lysine Residues but Is Constrained by Several MMD-Related RNF213 Mutations

RNF213 is an E3 ubiquitin ligase that can undergo autoubiquitination and also ubiquitinate various substrates [2,10,11]. Such ubiquitination activity is associated with its unique RING domain [11] as well as with its RZ domain that can, surprisingly, ubiquitinate even the lipid A moiety of Salmonella LPS [19].

**Figure 5:**
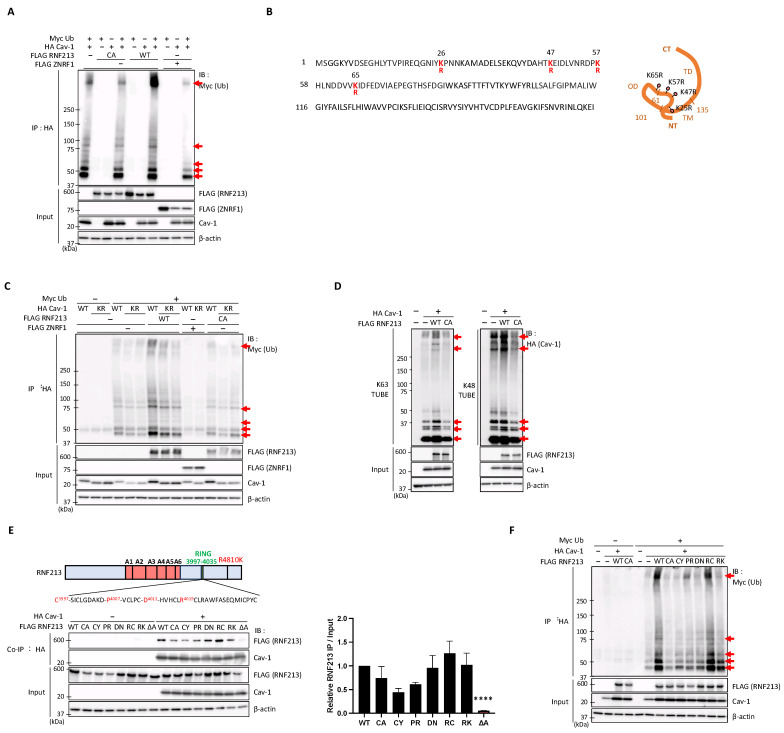
Ubiquitination of Cav-1 at multiple lysine residues by RNF213 and the effects of RNF213 MMD mutations. (**A**) Ubiquitination of Cav-1 in HEK293T cells overexpressing different combinations of FLAG-tagged RNF213 WT or mutant CA, HA-tagged Cav-1, and Myc-tagged ubiquitin (Ub). The extent of RNF213-mediated ubiquitination of Cav-1 was determined by immunoprecipitation (IP) with an anti-HA antibody and immunoblotting (IB) with the indicated antibodies. Arrows indicate ubiquitin-conjugated Cav-1. FLAG-tagged ZNRF1 was used as a control E3 ligase for Cav-1 ubiquitination. (**B**) Identification of lysine residues K26, K47, K57, and K65 in the N-terminal cytoplasmic region of Cav-1 as targets of RNF213-mediated ubiquitination (in Cav-1 sequence and image), as determined by LC-MS/MS comparisons of the effects of WT and CA mutant RNF213 (see Supplementary Tables S2 and S3). (**C**) Ubiquitination of Cav-1 was examined under the same conditions as in (**A**) but using WT Cav-1 or two independent Cav-1 KR mutants in which all four lysine (K) residues, identified by LC-MS/MS and shown in (**B**), were mutated to arginine (R) residues. Arrows indicate increased ubiquitin-conjugated Cav-1. (**D**) Tandem Ubiquitin-Binding Entity (TUBE) assays were performed using K63 and K48 TUBEs to determine the RNF213-mediated formation of K48- and/or K63-linked polyubiquitin chains on Cav-1. HEK293T cells overexpressing FLAG-tagged RNF213 WT or the CA mutant and HA-tagged Cav-1 were subjected to affinity pull-down with either K63 or K48 TUBEs, followed by IB with anti-HA antibody. Arrows indicate the increased K63- and K48-linked polyubiquitin chains formed on Cav-1 by WT RNF213. (**E**) (**Upper panel**) Schematic diagram of RNF213 and MMD mutations, with positions shown in red. (**Lower panel**) Co-immunoprecipitation (co-IP) experiments, to show the effects of MMD-associated RNF213 mutations on Cav-1 binding, were performed by overexpressing different combinations of FLAG-tagged WT or mutant RNF213 and HA-tagged Cav-1 in HEK293T cells, followed by co-IP with anti-HA antibody and IB with the indicated antibodies. β-actin was used as the protein loading control. (**Right panel**) The ratios of bound RNF213 to total input RNF213 were calculated (mean ± SED, *n* = 4), with the mean WT RNF213 ratio being arbitrarily defined as 1. An asterisk denotes a significant statistical difference from WT RNF213, determined by one-sample *t*-tests with Holm–Šídák correction for multiple comparisons (**** *p* < 0.0001). (**F**) Ubiquitination of Cav-1 was examined essentially as in (**A**) but using RNF213 WT and its various MMD-related mutations. Arrows indicate ubiquitin-conjugated Cav-1. All experiments were performed with a minimum of three independent experimental replicates to ensure consistency and reproducibility.

As RNF213 and Cav-1 interact intracellularly, we speculated that Cav-1 could potentially serve as a substrate of RNF213 and be directly ubiquitinated by its E3 ligase activity. We, therefore, transiently expressed combinations of tagged RNF213, Cav-1, and ubiquitin in HEK293T cells, performed immunoprecipitation (IP) experiments by pulling down HA-tagged Cav-1, and then detected the ubiquitinated products by immunoblot analysis. The results clearly demonstrate that the monomeric, and particularly the oligomeric, forms of Cav-1 (indicated by red arrows) exhibit enhanced polyubiquitination with WT RNF213 compared to the C3997A (CA) RNF213 mutation (Figure 5A). We also used FLAG-tagged ZNRF1, an E3 ubiquitin ligase that was previously reported to ubiquitinate Cav-1 and modulate its stability, thereby regulating Toll-like receptor (TLR) 4-triggered immune responses [58], as a positive control for Cav-1 ubiquitination. However, in our hands, we found no evidence that ZNRF1 promotes Cav-1 ubiquitination in HEK293T cells.

To identify specific residues on Cav-1 that serve as acceptors for RNF213-mediated polyubiquitin chain formation, we performed liquid chromatography (LC)–tandem MS (MS/MS) analysis using the WT and C3997A mutants of RNF213 to identify the ubiquitinated Cav-1 molecules. To ensure experimental reproducibility, we prepared four independent sets of WT and C3997A mutant samples (Supplementary Table S2). Through these repeated analyses, we identified lysine residues at positions K26, K47, K57, and K65 at the N-terminal cytoplasmic region of Cav-1 as ubiquitination targets of RNF213 (Figure 5B, Supplementary Table S3). To further validate the importance of these lysine residues in RNF213-mediated ubiquitination of Cav-1, we mutated all four K26, K47, K57, and K65 residues to arginine (R) residues (resulting in clones designated as Cav-1 KR) and then performed the IP ubiquitination assays with tagged RNF213, Cav-1, and ubiquitin. Only when WT RNF213 and WT Cav-1 were used in the assays was an enhanced pattern of monomeric and oligomeric Cav-1 ubiquitination observed, whereas two independently derived Cav-1 KR mutant clones displayed greatly reduced ubiquitination levels that were only slightly higher than samples lacking RNF213 or with the RNF213 C3997A mutation (Figure 5C).

Ubiquitination of Cav-1 by RNF213 suggests that RNF213 may target Cav-1 for proteasomal or lysosomal degradation or signal transduction pathways. Polyubiquitin chains attached to a substrate exhibit different regulatory properties depending on their characteristics. Polyubiquitin chains through Lys-48 (K48) linkage induce substrate degradation, whereas K63-linked polyubiquitin chains often function in signal transduction and DNA repair [59]. To identify the types of ubiquitination chains formed on Cav-1, we performed mass spectrometry and determined that RNF213 forms K29, K33, K48, and K63 ubiquitin chains on Cav-1 (Supplementary Table S4).

To confirm these MS/MS results, and to better understand the role of RNF213 in the ubiquitination of Cav-1, we co-expressed RNF213 and Cav-1 in HEK293 cells and employed a pull-down assay using Tandem Ubiquitin-Binding Entities (TUBEs) that specifically recognize specific linkages, such as K63 or K48 polyubiquitin chains [60]. This approach revealed that WT RNF213, but not its C3997A mutant, forms both K48- and K63-linked polyubiquitin chains (Figure 5D), and suggests that RNF213 may target Cav-1 for proteasomal degradation as well as signal transduction processes.

To date, numerous mutations in RNF213 of MMD patients have been reported [3,61]. We therefore sought to understand the functional consequences of several RING domain mutations, as well as the R4810K mutation, on the Cav-1 ubiquitination process. First, to analyze the effects of these mutations on Cav-1 binding, we used the RNF213 clones expressing the C3997Y, P4007R, D4013N, R4019C, or R4810K MMD patient mutations, as described previously [16]. Co-IP experiments with or without Cav-1 co-transfection demonstrated that although C3997Y and P4007R slightly reduce the binding affinities, none of the RNF213 mutations used significantly affect the ability of RNF213 to bind Cav-1 (Figure 5E).

Next, to examine the effects of these RNF213 mutations on Cav-1 ubiquitination, we performed the IP experiments with tagged ubiquitin as described above and found that, except for the R4019C mutation, all the other tested mutants, including R4810K, greatly reduce the level of Cav-1 ubiquitination compared to WT RNF213 (Figure 5F). Overall, these findings highlight the critical role of residues within and around the RING domain in mediating the ubiquitination of Cav-1 and imply that MMD-related RNF213 mutations may have a significant impact on Cav-1 degradation and/or Cav-1-mediated signal transduction processes.

Furthermore, using the Tyr14 phosphomimetic (YD) and phosphodefective (YF) Cav-1 mutants, we observed no significant effect on the ubiquitination of Cav-1 (Supplementary Figure S2A), similar to the lack of effect of these mutants on Cav-1 binding to RNF213 (Supplementary Figure S1B).

### 3.6. RNF213 Knockdown Enhances Cav-1 Y14 Phosphorylation Under H_2_O_2_ and Affects Nitric Oxide Stimulation in HPAECs

**Figure 6:**
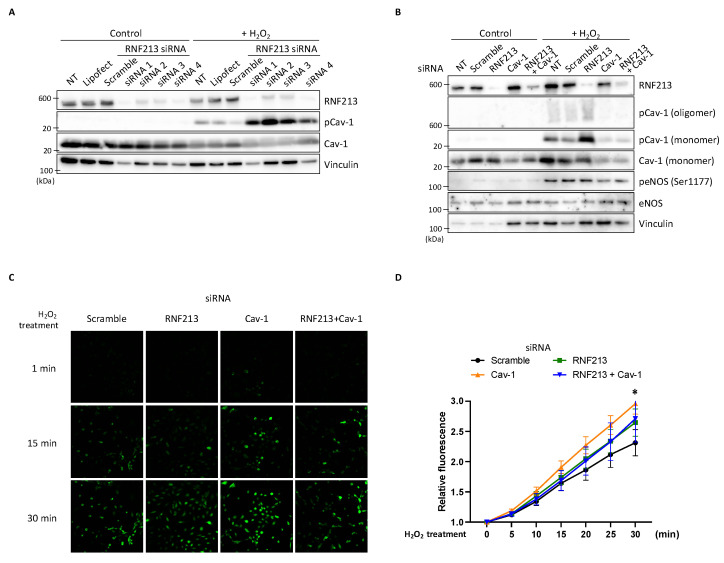
RNF213 knockdown enhances Cav-1 Y14 phosphorylation under H_2_O_2_ and affects nitric oxide stimulation in HPAECs. (**A**) Immunoblot analysis illustrating the effects of hydrogen peroxide (H_2_O_2_) and RNF213 knockdown on endogenous monomeric phosphorylated Y14 Cav-1 (pCav-1) levels in HPAECs. Cells were non-transfected (NT) or transfected with scramble or four different RNF213-targeted siRNAs, and then untreated (control) or treated with 1 mM H_2_O_2_ for 30 min to induce oxidative stress and lead to observable levels of monomeric pCav-1. Vinculin was used as a protein loading control. (**B**) Immunoblot analysis, used to investigate monomeric and oligomeric pCav-1 levels in HPAECs in response to H_2_O_2_, was essentially performed as in (**A**), but with siRNAs targeting RNF213 (siRNA 2), Cav-1, or both RNF213 and Cav-1, together with the indicated antibodies. (**C**) Fluorescence microscopy images of HPAECs incubated with DAF-FM DA dye for determining relative nitric oxide (NO) bioavailability. Cells were transfected with siRNAs targeting RNF213 (siRNA 2), Cav-1, or both RNF213 and Cav-1 and treated with H_2_O_2_ for 30 min as in (**A**). Photographs of sample cells were taken at 1, 15, and 30 min after treatment. (**D**) Quantitative fluorescence intensity of the NO production over time, determined in (**C**). Raw fluorescence intensity values for DAF-FM were measured every 5 min and normalized to the value at 1 min for each condition. Data are shown as means ± SEM (*n* = 7). Statistical comparisons between groups at each time point were performed using two-way ANOVA (* *p* < 0.05). All analyses involving HUVECs and HPAECs were performed using a single donor-derived lot for each cell type, with at least three independent experimental replicates for (**A**,**C**,**D**), and with two independent experimental replicates and three technical replicates for (**B**).

In our initial studies, we consistently observed that down-regulation of RNF213 by RNF213-targeted siRNA in HPAECs resulted in very faint but discernable increases in the Cav-1 Y14 phosphorylation levels. To better visualize these Cav-1 phosphorylation levels, we treated the HPAECs with hydrogen peroxide (H_2_O_2_), which had previously been shown to elevate the levels of Cav-1 phosphorylation [62]. Indeed, upon H_2_O_2_ treatment, we found an observable stress-induced activation of Cav-1 Y14 phosphorylation in non-transfected (NT), Lipofectamine-only, and scramble siRNA controls compared to the H_2_O_2_-untreated cells. However, knockdown of RNF213 with any one of four different RNF213 siRNAs resulted in dramatic increases in the Cav-1 Y14 phosphorylation with H_2_O_2_ (Figure 6A). Repeated experiments with untreated and H_2_O_2_-treated HPAECs transfected with siRNAs targeting RNF213, Cav-1, or both RNF213 and Cav-1 clearly demonstrated enhanced levels of both the monomeric and oligomeric forms of phosphorylated Cav-1 Y14 following RNF213 knockdown but almost a complete loss of these signals when Cav-1 was knocked down alone or in combination with RNF213. Furthermore, whereas phosphorylated (at Ser1177) endothelial nitric oxide synthase (eNOS) levels were elevated by H_2_O_2_ [63], there was no effect of RNF213 or Cav-1 knockdown on the total or phosphorylated eNOS levels (Figure 6B). These results clearly demonstrate that the presence of RNF213 specifically suppresses the phosphorylation of Cav-1 Y14 in both its monomeric and oligomeric structural states.

In endothelial cells, Cav-1 is known to bind eNOS and maintain it in an inactive state. After eNOS release from Cav-1 and activation, the synthesized nitric oxide (NO) stimulates Cav-1 Y14 phosphorylation [64] to regulate eNOS-derived NO through a negative-feedback mechanism and control vascular homeostasis [65]. We, therefore, questioned whether the increased phosphorylated Cav-1 levels observed in response to RNF213 down-regulation under H_2_O_2_ conditions, which have been shown to increase NO release in endothelial cells [63], would alter the NO levels and help explain the role of RNF213 in vascular control. We therefore quantified NO production in H_2_O_2_-treated HPAECs using the NO-sensitive fluorescent dye, DAF-FM DA. A general increase in DAF-FM fluorescence signal was observed over a 30 min period in the H_2_O_2_-treated HPAECs, indicative of the immediate production of NO. The knockdown of Cav-1 resulted in significant increases in the DAF-FM signal compared to the scramble control, confirming the expected increase in NO synthesis presumably resulting from eNOS release and activation [66,67]. Importantly, the concurrent knockdown of RNF213 and Cav-1 attenuated this increase in NO to levels equivalent to those of the RNF213 knockdown and scramble, suggesting a potential interaction between RNF213 and Cav-1 in regulating the NO signal pathway (Figure 6C,D).

## 4. Discussion

At the core of this study’s findings is the functional interaction between RNF213 and one of its few mammalian substrates identified to date, Cav-1. The relationship between these two proteins is initiated by the ATP-dependent binding of Cav-1 to a region encompassing the two functional AAA+ domains of RNF213. This interaction not only modifies mutant RNF213-induced inflammatory and apoptotic signaling events but also allows RNF213 to function as a molecular switch that can either ubiquitinate or phosphorylate key residues of Cav-1, depending on RNF213 activity and the cellular energy and stress status. Based on the pivotal roles that RNF213 and Cav-1 play in various cellular events, these findings argue for a mechanism that is both complex and potentially diverse in its physiological outcomes.

The E3 ligase activity of RNF213, dependent on its RING domain and RZ finger, is recognized as central to its core functionality [13,68], but its ability to ubiquitinate Cav-1 adds an additional level of complexity to its role in cellular signaling. Cav-1 is known to be ubiquitinated at several of its N-terminal lysine residues, namely K5, K26, K30, K39, K47, and K57, for trafficking Cav-1 from early to late endosomes for lysosomal-mediated degradation [69], and solely at K39 by the E3 ligase ZNRF1 or at K86 by nitric oxide stimulation [58] for proteasomal degradation of Cav-1. However, our finding that RNF213 ubiquitinates Cav-1 only at the cytoplasmic sites K26, K47, K57, and K65 implies that while these modifications may allow trafficking of Cav-1, they may not necessarily be involved in proteasomal-directed Cav-1 degradation. Indeed, we found no evidence that RNF213 promotes Cav-1 degradation. Nevertheless, our studies also determined that RNF213 catalyzes both K48 and K63 polyubiquitin linkages as well as possible K29 linkages on Cav-1, revealing the potential complexity of Cav-1 regulation. K48 linkages are primarily associated with degradation through the 26S proteasome system [70], suggesting that RNF213 may still target Cav-1 for degradation, possibly under specific conditions to regulate cellular responses to stress or signaling. In contrast, K63 linkages are often involved in modulating protein–protein interactions and activating downstream signaling cascades, such as autophagy [71] and NF-κB signaling [72], indicating that RNF213 may also regulate the involvement of Cav-1 in such key cellular processes. Similarly, K29-linked chains appear to function in signaling, including the Wnt/β-catenin pathway and the regulation of protein kinase activity, as well as in autophagy [71,73]. Several earlier studies have already demonstrated that RNF213 is able to form various types of ubiquitin chains, including M1 [74], K11 [45], K48 [18], and K63 [16,17,20], underscoring its diverse functions in both proteolytic and non-proteolytic processes. To better understand the functional significance of such Cav-1 ubiquitination patterns, further examination is required of the specific E2-conjugating enzymes, such as UBE2D2, UBE2L3, or UBE2N/V1, which have only been shown to function in RNF213 auto-ubiquitination [11,16,45], and how these may fine-tune its degradation and downstream signaling events.

As expected, the RNF213 RING domain mutations C3997Y, P4007R, and D4013N, as well as the MMD-associated R4810K mutant that has been shown to affect its E3 ligase activity [11], display considerably lower levels of Cav-1 ubiquitination than WT RNF213, and levels comparable to that of the RING-dead mutant C3997A. As none of these mutants significantly reduce RNF213 to Cav-1 binding, our findings confirm that it is primarily the E3 ligase activity of the RING domain that determines Cav-1 ubiquitination and that such changes in Cav-1 ubiquitination may be related to MMD pathogenesis. Whether the RZ finger is also involved in Cav-1 ubiquitination, and thus is also a factor in MMD development, remains to be determined.

Apart from the E3 ligase activity of RNF213, two other essential requirements are necessary for effective Cav-1 ubiquitination: a physical interaction between RNF213 and Cav-1, and the energy status of the cell. That Cav-1 binds RNF213 only within the two (of six) AAA+ ATPase domains, namely the third (A3) and fourth (A4) domains, which can bind ATP through their Walker A (P-loop) regions and also have catalytic ATPase activity as a function of their Walker B regions [68], is of functional significance. Recent studies have demonstrated the importance of these AAA+ A3 and A4 domains in RNF213 functionality. For example, the Walker A and Walker B regions of both A3 and A4 are needed to target RNF213 to lipid droplets [41]; the Walker A regions of A3 and A4 and the Walker B region of A4, but not of A3, are necessary for ubiquitination of the lipid moiety of bacterial LPS [19]; and the Walker A and Walker B regions of A3 are essential for mutant RNF213-induced NF-κB signaling and apoptosis [16]. We similarly find that the A3 domain of RNF213 is essential for binding Cav-1, as the deletion of A3 from either WT RNF213 or from the highly interactive N2 fragment of RNF213 completely eliminates their interactions with Cav-1. Furthermore, residues critical for ATP binding within the Walker A region of A3 are found essential for binding as mutations in these residues, but not in those in the Walker A of A4 or in the Walker B of A3 and A4, drastically reduce the RNF213 and Cav-1 interaction. These results suggest to us that Cav-1, rather than directly binding to the Walker A domain, requires RNF213 to be in an ATP-bound state for binding, and, indeed, ATP binding to the Walker A of many AAA+ ATPases is a prerequisite for substrate binding [75]. We further demonstrate the need for ATP in this process by modifying intracellular ATP availability via the inhibition of glycolysis and oxidative phosphorylation. The reduced Cav-1 binding observed under severely depleted ATP conditions, and the recovery of the interactions with increasing cellular ATP levels, suggest that the Cav-1 and RNF213 interaction is highly sensitive to the energy status of the cell. It may be expected, therefore, that under low ATP levels, such as during hypoxia [76], this reduced binding could limit Cav-1 ubiquitination and thereby modify downstream signaling events. In support of this, the interaction between the AAA+ ATPase NVL2 and its substrate DOB1 was abolished by the depletion of ATP with hexokinase and D-glucose [77]. In addition to enhancing Cav-1 binding, other studies have shown that ATP binding to the A3 and A4 domains of RNF213 increases its E3 ligase activity and is essential for ubiquitin transfer [68], and that substrate binding stimulates the ATPase activity needed to promote substrate release upon ATP hydrolysis in other AAA+ ATPases [78,79]. Based on these findings, we propose a simple model in which, under a favorable energy state, ATP binding at the Walker A of A3 promotes a conformational change within RNF213, possibly oligomerization [13,80], that allows Cav-1 to bind within its A3 and A4 domains and to stimulate its RING E3 ligase activity so as to enhance Cav-1 ubiquitination and, through enhanced ATP hydrolysis, to subsequently release the modified Cav-1.

However, our findings not only suggest that Cav-1 serves as a substrate for RNF213 ubiquitination, but also that Cav-1 functions as a negative regulator of RNF213-mediated processes. Our previous studies determined that several RNF213 RING mutations could increase NF-κB activation as well as apoptosis, from which we concluded that these RING mutations serve a gain-of-function role [16]. In our current study, we used the same reporter system and confirmed that while RNF213 RING mutations similarly elevate NF-κB activation and apoptosis, co-expression of Cav-1 completely ameliorates these increased levels. More recently, tyrosine phosphorylation of RNF213 has been shown to enhance its oligomerization and the subsequent activation of the RZ finger, which then is able to ubiquitinate CYLD/SPATA2 and lead to their degradation and subsequent NF-κB activation [21]. RNF213 RING mutants were shown to serve as RZ finger gain-of-function mutations and to further impact this NF-κB activation. The exact mechanism by which Cav-1 expression represses mutant RNF213-mediated NF-κB activation is unclear; however, it is possible that binding of Cav-1 to the RNF213 AAA+ domain modifies its activity, possibly through the RZ finger, and alters downstream events to limit NF-κB activation and apoptosis induced by the RING mutations. Indeed, the activation of NF-κB and apoptosis by these mutations has been shown to be AAA- and ATPase-dependent [16,21].

The binding of Cav-1 to its protein partners is often considered to be mediated by an interaction between its caveolin-scaffolding domain (CSD) and the aromatic-rich caveolin-binding motif (CBM) on specific regulatory molecules, such as Src-family kinases, receptor tyrosine kinases, and eNOS, which are then regulated by Cav-1 [81]. Other studies, however, have demonstrated that Cav-1 and target protein interactions do not necessarily involve either of these regions [81], and, similarly, in our current study, mutations or deletion of the Cav-1 CSD, or of the two putative RNF213 CBMs that we identified, did not alter the interaction between Cav-1 and RNF213. This then presumably allows Cav-1 to bind RNF213 through its two AAA+ domains and concomitantly its other regulatory partner(s), such as eNOS, through its CSD. In the case of eNOS, which is predominantly found in the caveolae of endothelial cells, where it catalyzes the production of nitric oxide (NO), a key regulator of blood pressure, vascular tone, and angiogenesis, binding to the CSD of Cav-1 stabilizes eNOS and maintains it in an inactive state, thereby limiting NO generation [82]. Depending on the signal, which may or may not involve Ca^2+^, calmodulin displaces eNOS from Cav-1, thereby activating eNOS and resulting in an initial phase of NO release [83]. Further modification of the eNOS S1177 and T495 phosphorylation sites, such as by H_2_O_2_, then sets the activation state of eNOS and results in prolonged NO release [84]. To limit the detrimental effects of excessive NO, a feedback system comes into play where elevated NO activates Src that then phosphorylates Cav-1 Y14. This alters the Cav-1 CSD configuration to enhance a stronger binding to eNOS, thereby inactivating eNOS and reducing NO levels while increasing Cav-1 trafficking and caveolae release from the plasma membrane [85]. In our study, we observed that Cav-1 knockdown resulted in increased NO production, even though phosphorylation of eNOS at S1177 remained unchanged. Previous studies have shown that phosphorylation at T495 negatively regulates eNOS activity by reducing NO production [86]. Although we did not assess T495 phosphorylation in this study, it is possible that Cav-1 knockdown affected this inhibitory site, leading to enhanced eNOS activity and increased NO levels. Another plausible explanation is that the NO release we observed occurred during the initial phase of NO’s release before the activation phase, when S1177 and T495 are phosphorylated and NO production is enhanced and prolonged. Our observation that RNF213 knockdown in the presence of H_2_O_2_ specifically enhances Cav-1 Y14 phosphorylation, without affecting total Cav-1 levels or the phosphorylation of eNOS S1177, argues that RNF213 may normally inhibit the phosphorylation of Cav-1, for example by simply limiting Src access to Cav-1 when bound by RNF213, modifying the intracellular location of Cav-1 away from caveolae, or promoting the trafficking or degradation of Src through ubiquitination. Knockdown of RNF213 may then alleviate this inhibition and allow Src to phosphorylate Cav-1. Intriguingly, it appears that it is the Src family member FYN, and not Src itself, that is activated by H_2_O_2_ and phosphorylates Cav-1 Y14 under these stress conditions [87] and that, similar to Src, S-nitrosylation of FYN can promote its phosphorylation [88] and engage it in the feedback system. Furthermore, the significant increase in NO generation following Cav-1 knockdown under H_2_O_2_ conditions is as would be expected from the release of the sequestered eNOS from Cav-1 binding and the lack of feedback control. However, we also found a reduction in these increased NO release levels following additional RNF213 knockdown to levels equivalent to the base NO levels. Cav-1 expression levels have been estimated to be 200-fold higher than eNOS in human endothelial cells [30], and H_2_O_2_ enlarges the Cav-1 pool even further. Therefore, despite the knockdown of Cav-1, the available Cav-1 pool could still be sufficient to serve as a target for RNF213-associated phosphorylation that would allow the phosphorylated Cav-1 to re-bind eNOS more tightly, curtail its activity, and lead to a net decrease in NO synthesis.

The findings of this study not only provide a new understanding of the functional regulation of RNF213 and Cav-1 and their effects on NO release in endothelial cells, but also give insights into how disruption of these processes by MMD-related mutations, such as R4810K, can lead to abnormal signaling events that contribute to endothelial cell dysfunction, altered vascular remodeling, and MMD pathogenesis. For example, potential changes to the extent and configuration of the Cav-1 K48 and K63 ubiquitination chains by the mutations could disrupt the regulatory balance between protein degradation, signal transduction, and cellular energetics, and lead to a dysregulated Cav-1 pool [35,89] that impairs its role in maintaining endothelial cell integrity and vascular function or lead to altered inflammatory or apoptotic responses [21] that promote the vascular occlusion observed in MMD patients. Furthermore, by functioning as loss-of-function mutations, they may increase the level of Cav-1 phosphorylation, thereby disrupting the normal RNF213 control of NO bioavailability and contributing to changes in vascular tone and the development of arterial stenosis and ischemic events found in MMD patients. In addition, the potential link between ATP availability and RNF213-mediated ubiquitination also raises important questions about how metabolic stress or altered energy homeostasis might influence vascular function. For instance, in pathological conditions such as diabetes, ischemia, or inflammation, disruptions in cellular metabolism could impair the activity of RNF213 and its ability to regulate Cav-1, leading to endothelial dysfunction and promoting vascular abnormalities. Clearly, a deeper understanding of such relationships and pathways will be crucial for developing targeted interventions that can restore endothelial cell function and improve outcomes for patients with RNF213-related vasculopathies. To achieve this, future studies using endogenous expression systems, such as CRISPR-mediated knockins in endothelial cells, will be necessary to confirm that the interactions and ubiquitination patterns we observed under overexpression in HEK293T cells, which may elevate RNF213 and Cav-1 levels above their physiological levels, also occur under physiologically relevant expression conditions.

Finally, it has not escaped our notice that many of the key functions associated with RNF213, such as lipid metabolism, lipid droplet association, tumor development, and bacterial and viral endocytosis and regulation, are also features of Cav-1 functionality, and so it would not be unexpected to find these processes to be also mediated by this RNF213–Cav-1 axis.

## Data Availability

The data that support the findings of this study are available from the author, J.C., upon reasonable request.

## Supplementary Materials

The following supporting information can be downloaded at https://www.mdpi.com/article/10.3390/cells14110775/s1, Figure S1: Co-immunoprecipitation (co-IP) experiment to determine the RNF213 and Cav-1 domains necessary for their interaction; Figure S2: Co-immunoprecipitation (co-IP) ubiquitination assays to determine the effects of Cav-1 Y14 phosphorylation status on Cav-1 binding to RNF213; Table S1: Primary antibodies used in this study; Table S2: Identification of peptide fragments and ubiquitination of Cav-1 protein by mass spectrometry; Table S3: Difference in ubiquitinated lysine residues ratio of Cav-1 protein between WT RNF213 and CA mutant-transfected cells; Table S4: Types of ubiquitination chain in Cav-1 protein.
