# Supplementary material for: RNF213 Acts as a Molecular Switch for Cav-1 Ubiquitination and Phosphorylation in Human Cells
Source: Cells. 2025 May 25;14(11):775. doi: 10.3390/cells14110775 (PMC12153744; doi:10.3390/cells14110775)
Supplement: Supplementary file 1 [file cells-14-00775-s001.zip › cells-3569371-supplementary.pdf]

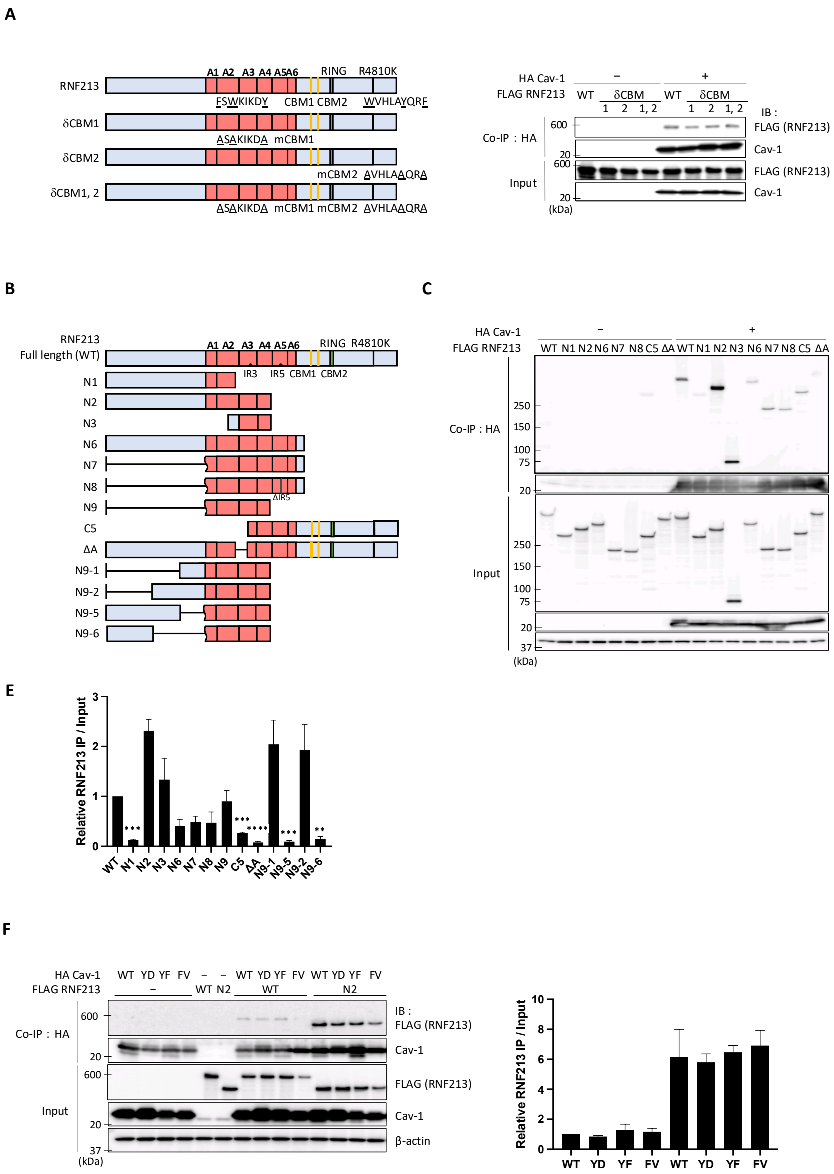

Supplementary Figure S1. Co-immunoprecipitation (Co-IP) experiments to determine the RNF213 and Cav-1 domains and motifs necessary for their interaction. (A) Schematic diagram of WT RNF213 and its two putative Cav-1 binding motifs (CBMs: CBM1, FSWKIKDY and CBM2, WVHLAYQRF) and their mutated forms (mCBM1 and mCBM2) in which the aromatic amino acids (underlined) are mutated to A residues in either δCBM1, δCBM2 or both δCBM1,2. HEK293T cells were co-transfected with these FLAG-tagged WT or the CBM-mutated RNF213 plasmids together with or without HA-tagged Cav-1 plasmids as indicated. Interactions between RNF213 and Cav-1 were detected by co-IP with anti-HA antibody followed by immunoblotting (IB) with the indicated antibodies. (B) Schematic diagram of full-length WT RNF213 and its various truncated forms used for Co-IP experiments. (C) Co-IP experiments in HEK293T cells overexpressing FLAG-tagged WT RNF213 or various truncated RNF213 fragments with or without HA-tagged Cav-1, and co-IP with anti-HA antibody. (D) Co-IP experiments in HEK293T cells overexpressing FLAG-tagged WT RNF213 or various deleted RNF213 fragments with or without HA-tagged Cav-1, and co-IP with anti-HA antibody and IB with the indicated antibodies. (E) The ratios of bound RNF213 to total input RNF213 were calculated (mean±SED, *n*=3), with the mean WT RNF213 ratio being arbitrarily defined as 1. Asterisk denotes significant statistical difference from WT RNF213, determined by one-sample t-tests with Holm–Šídák correction for multiple comparisons (**p < 0.01, ***p < 0.001, and ****p < 0.0001). (F) Co-IP experiments in HEK293T cells overexpressing FLAG-tagged WT or N2 RNF213 with HA-tagged

WT Cav-1 or carrying Cav-1 Y14 phosphomimetic (YD) or phosphodefective (YF) point mutations, or with mutations in the Caveolin Scaffold Domain (CSD, FV) followed by co-IP with anti-HA antibody and IB with the indicated antibodies. (Right panel) The ratios of bound RNF213 to total input RNF213 were calculated, with the mean WT RNF213 ratio being arbitrarily defined as 1. No effects on binding were observed by changes in the Cav-1 phosphorylation status or mutations in the caveolin scaffold domain. For all immunoblots, β-actin was used as protein loading control. In (A), (C), (D) and (E) experiments were performed with at least three independent experimental replicate and in (F) experiments were performed with two independent experimental replicates and three technical replicates.

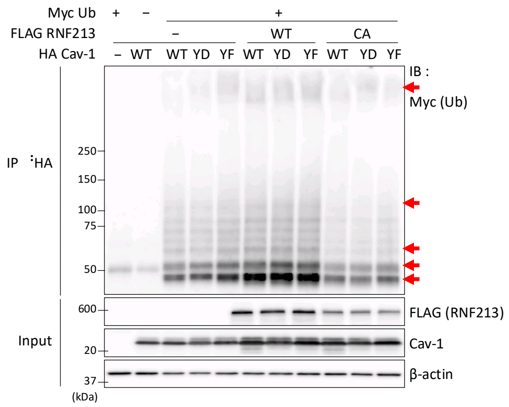

Supplementary Figure S2. The effects of Cav-1 Y14 phosphorylation status on Cav-1 binding to RNF213. HEK293T cells were co-transfected with combinations of Myc-tagged ubiquitin (Ub), FLAG-tagged WT or CA mutant RNF213, and HA-tagged WT Cav-1 or the phosphomimetic (YD) and phosphodefective (YF) Cav-1 mutations as indicated, followed by immunoprecipitation (IP) with anti-HA antibody and immunoblotting (IB) with anti-Myc antibody. Arrows show ubiquitin-conjugated Cav-1. Experiments were performed with three independent experimental replicates to ensure consistency and reproducibility.

Supplementary Table S1. Primary antibodies used in this study.

Antibody	Dilution Immunoblotting	Dilution Cell imaging	Clone	Source
rabbit polyclonal anti-RNF213	1:8000	1:2000	ABC1391	Millipore
mouse monoclonal anti-Cav-1		1:300	sc-53564	Santa Cruz Biotechnology
mouse monoclonal anti-Cav-1	1:10000	1:5000	66067-1	Proteintech
rabbit monoclonal anti-Cav-1	1:4000		3238	Cell Signaling Technology
rabbit polyclonal anti-Phospho-Caveolin-1 (Tyr14)	1:4000		3251	Cell Signaling Technology
rabbit monoclonal anti-HA	1:4000		3724	Cell Signaling Technology
mouse monoclonal anti-FLAG M2	1:4000		F3165	Sigma-Aldrich
mouse monoclonal anti-c-Myc	1:1000		sc-40	Santa Cruz Biotechnology
rabbit monoclonal anti-Cleaved-Caspase-3	1:4000		9664	Cell Signaling Technology
rabbit polyclonal anti-Phospho-eNOS (Ser1177)	1:4000		9571	Cell Signaling Technology
rabbit monoclonal anti-eNOS	1:4000		32027	Cell Signaling Technology
rabbit monoclonal anti-β-Actin	1:4000		13E5	Cell Signaling Technology

Supplementary Table S2. Identification of peptide fragments and ubiquitination of Cav-1 protein by mass spectrometry.

No.	RT [min]	m/z [Da]	MH+ [Da]	Charge	Annotated peptide fragment sequence	Position	Type	Ubiquitination site	Modifications
1	58.4752	734.3778	2934.4894	+4	YVDSEGHLYTVPIREQGNI-YKPNNK	6-30	Normal	-	-
2	58.2377	762.8794	3048.4958	+4	YVDSEGHLYTVPIREQGNI-YkPNNK	6-30	Ubiqui-tinated	26	K21(GlyGly)
3	19.0952	435.5591	1304.6626	+3	EQGNIYKPNNK	20-30	Normal	-	-
4	22.6528	473.5727	1418.7035	+3	EQGNIYkPNNK	20-30	Ubiqui-tinated	26	K7(GlyGly)
5	20.1799	652.8348	1304.6623	+2	EQGNIYKPNNK	20-30	Normal	-	-
6	22.5943	709.8555	1418.7037	+2	EQGNIYkPNNK	20-30	Ubiqui-tinated	26	K7(GlyGly)
7	48.7979	570.5311	2279.1024	+4	EQGNIYKPNNKAMADELSEK	20-39	Normal	-	-
8	47.8904	599.0411	2393.1425	+4	EQGNIYKPNNkAMADELSEK	20-39	Ubiqui-tinated	30	K11(GlyGly)
9	46.2717	484.7331	1935.9104	+4	AMADELSEKQVYDAHTK	31-47	Normal	-	-
10	47.1323	513.2443	2049.9555	+4	AMADELSEkQVYDAHTK	31-47	Ubiqui-tinated	39	K9(GlyGly)
11	38.6636	488.7329	1951.9099	+4	AmADELSEKQVYDAHTK	31-47	Normal		M2(Oxidation)
12	40.6619	517.2437	2065.9530	+4	AmADELSEkQVYDAHTK	31-47	Ubiqui-tinated	39	M2(Oxidation); K9(GlyGly)
13	44.3538	645.9753	1935.9113	+3	AMADELSEKQVYDAHTK	31-47	Normal	-	-
14	47.1865	683.9909	2049.9582	+3	AMADELSEkQVYDAHTK	31-47	Ubiqui-tinated	39	K9(GlyGly)
15	38.4674	651.3061	1951.9037	+3	AmADELSEKQVYDAHTK	31-47	Normal		M2(Oxidation)
16	40.6507	689.3223	2065.9522	+3	AmADELSEkQVYDAHTK	31-47	Ubiqui-tinated	39	M2(Oxidation); K9(GlyGly)
17	45.7762	714.3721	2141.1017	+3	QVYDAHTKEIDLVNRDPK	40-57	Normal	-	-
18	46.9077	752.3876	2255.1484	+3	QVYDAHTkEIDLVNRDPK	40-57	Ubiqui-tinated	47	K8(GlyGly)
19	52.1789	707.0437	2119.1166	+3	EIDLVNRDPKHLNDDVVK	48-65	Normal	-	-
20	53.0811	745.0602	2233.1662	+3	EIDLVNRDPkHLNDDVVK	48-65	Ubiqui-tinated	57	K10(GlyGly)
21	81.738	665.9256	3325.5989	+5	HLNDDVVKID-FEDVIAEPEGTHSFDGIWK	58-86	Normal	-	-
22	95.0449	688.7354	3439.6480	+5	HLNDDVVkID-FEDVIAEPEGTHSFDGIWK	58-86	Ubiqui-tinated	65	K8(GlyGly)
23	76.6184	832.1523	3325.5873	+4	HLNDDVVKID-FEDVIAEPEGTHSFDGIWK	58-86	Normal	-	-
24	81.4184	860.6657	3439.6410	+4	HLNDDVVkID-FEDVIAEPEGTHSFDGIWK	58-86	Ubiqui-tinated	65	K8(GlyGly)

Supplementary Table S3. Difference in ubiquitinated lysine residues ratio of Cav-1 protein between WT RNF213 and CA mutant-transfected cells.

Ubiquitination site	Sequence	Modifications	Area (ratio; WT/CA)				Mean
			Sample 1	Sample 2	Sample 3	Sample 4	
26	EQGNIYKPNNK	K7(GlyGly)	1.65	1.38	1.78	1.39	1.55
26	EQGNIYKPNNK	K7(GlyGly)	1.81	1.41	2.10	1.29	1.65
26	YVDSEGHLYTVPIREQGNIYKPNNK	K21(GlyGly)	1.00	1.00	1.00	1.00	1.00
30	EQGNIYKPNNKAMADELSEK	K11(GlyGly)	1.16	1.21	0.46	1.00	0.96
39	AMADELSEKQVYDAHTK	K9(GlyGly)	0.73	0.50	0.41	1.00	0.66
39	AmADELSEKQVYDAHTK	M2(Oxidation); K9(Gly-Gly)	1.41	0.46	0.23	2.42	1.13
39	AMADELSEKQVYDAHTK	K9(GlyGly)	0.63	0.47	0.36	1.00	0.62
39	AmADELSEKQVYDAHTK	M2(Oxidation); K9(Gly-Gly)	0.95	0.32	0.28	2.67	1.06
47	QVYDAHTKEIDLVNRDPK	K8(GlyGly)	2.14	2.05	0.94	1.00	1.53
57	EIDLVNRDPKHLNDDVVK	K10(GlyGly)	1.00	1.55	1.06	1.00	1.15
65	HLNDDVVKID-FEDVIAEPEGTHSFDGIWK	K8(GlyGly)	1.00	1.00	1.00	1.00	1.00
65	HLNDDVVKID-FEDVIAEPEGTHSFDGIWK	K8(GlyGly)	1.00	1.00	9.83	1.00	3.21

The red text indicates lysine residues that showed an increased ubiquitination ratio in WT compared to the CA mutation.

Supplementary Table S4. Types of ubiquitination chain in Cav-1 protein.

Table .	Specific peptide sequence of ubiquitin chain	RT (min)	m/z [Da]	charge	Area (ratio: WT/CA)				Mean
					Sample 1	Sample 2	Sample 3	Sample 4	
K6	M(ox)QIFVK(GG)TLTGK		465.9270	+3					
	MQIFVK(GG)TLTGK		690.3895	+2					
	MQIFVK(GG)TLTGK		460.5954	+3					
K6+tag	LISEED-LNGEFM(ox)QIFVK(GG)TLTGK		881.4510	+3					
	LISEED-LNGEFM(ox)QIFVK(GG)TLTGK		661.3401	+4					
	LISEEDLNGEFMQIFVK(GG)TLTGK		876.1193	+3					
	LISEEDLNGEFMQIFVK(GG)TLTGK		657.3413	+4					
K11	TLTGK(GG)TITLEVEPSDTIENVK	68.15	801.4269	+3	0.32				0.32
	TLTGK(GG)TITLEVEPSDTIENVK	68.15	601.3220	+4	0.32				0.32
K27	TITLEVEPSDTIENVK(GG)AK		701.0390	+3					
	TITLEVEPSDTIENVK(GG)AK		526.0311	+4					
K29	AK(GG)IQDK	22.92	408.7324	+2	0.37	4.63	1.25	1.49	1.93
K33	IQDK(GG)EGIPPDQQR	30.16	546.6130	+3	4.47				4.47
K48	LIFAGK(GG)QLEDGR	51.57	487.6001	+3	3.47	1.75	1.77	1.62	2.15
K63	TLSDYNIQK(GG)ESTLHLVLR	68.21	561.8050	+4	3.23	5.26	2.75		3.75
	TLSDYNIQK(GG)ESTLHLVLR	68.21	748.7376	+3	2.88	3.64	2.71		3.08
M1	GGM(ox)QIFVK		448.2390	+2					
	GGMQIFVK		440.2415	+2					

The red text highlights the ubiquitination sites of Cav-1 that were identified as highly altered between WT RNF213 and the CA variant. Blank cells in the "Area" column indicate that the modifications were not detected.
